# Heterogeneity in valvular heart disease: differential predictive performance of CAR

**DOI:** 10.1097/JS9.0000000000003471

**Published:** 2025-09-11

**Authors:** Xing-Ji Gong, Shou-Meng Geng, Yong-Tao Zhang

**Affiliations:** a Department of Emergency Internal Medicine, The Affiliated Hospital of Qingdao University, Qingdao, Shan dong, China; b Department of Cardiology, The Affiliated Hospital of Qingdao University, Qingdao, Shan dong, China

*Dear Editor,*

We appreciate the authors’ work in the recent article “Prognostic Value of C-reactive Protein to Albumin Ratio in Patients with Valvular Heart Disease: Nationwide Prospective Cohort Study”^[1]^.The study proposes integrating the C-reactive protein-to-albumin ratio (CAR) into a multimodal risk assessment model, combining it with NT-proBNP and imaging techniques (e.g., echocardiography, cardiac MRI) to enhance risk stratification accuracy and guide preoperative nutritional/anti-inflammatory interventions. This approach offers a scientifically robust and clinically practical biomarker strategy, addressing the current gap in nutritional and inflammatory assessments within existing guideline. Building upon this foundation, we offer the following insights for discussion. In accordance with the TITAN 2025guideline, we ensured transparency in the preparation of this article^[2]^.

Perspective 1: The Revolutionary Value of CAR as a Novel Inflammatory-Nutritional Composite Biomarker in VHD Prognosis. This study is the first to systematically validate the prognostic value of the C-reactive protein-to-albumin ratio (CAR) in a large-scale VHD cohort (*n* = 5519), with innovations demonstrated across three dimensions:Breakthrough in Pathophysiological Mechanism: Traditional biomarkers like NT-proBNP solely reflect hemodynamic stress, whereas CAR simultaneously captures two core pathological processes – inflammation (CRP) and nutritional status (albumin). Data reveal significant correlations between CAR and NT-proBNP (*r* = 0.32) as well as liver function markers (direct bilirubin, *r* = 0.14), indicating its ability to reflect the synergistic effects of cardiogenic hepatic congestion, systemic inflammation, and protein-energy wasting. This multidimensional assessment aligns perfectly with the “cardio-hepatic-renal” comorbidity pattern common in VHD patients.Clinical Advantages: In ROC curve analysis, CAR’s AUC significantly outperformed standalone CRP or albumin^[2]^, with a threshold of 0.15 achieving 56% sensitivity and 73% specificity. Notably, CAR provided incremental value to traditional risk models (NRI = 0.57), particularly for multivalvular disease (MVHD, aHR 1.50) and conservatively managed subgroups (2-year mortality 14.9%). Its “dual-assessment” feature makes it ideal for rapid screening in resource-limited settings.Therapeutic Implications: High CAR (>0.15) patients exhibited residual mortality risk (2.1%) post-surgery, suggesting anatomical correction alone may not reverse the inflammation-malnutrition vicious cycle. This supports a combined “preoperative nutrition/anti-inflammatory intervention + surgery” management paradigm.

Perspective 2: Heterogeneity in VHD and Its Implications for Biomarker Selection. The study highlights critical biomarker-response variations: Mitral Stenosis (MS) Specificity: CAR lacked prognostic value in MS (*P* = 0.544), likely due to this subgroup’s unique characteristics: youngest mean age (55.9 years), 93.5% rheumatic etiology, and early surgical intervention. Their inflammation may be controlled by antibiotics/anti-rheumatic therapy, while hemodynamic stress (e.g., left atrial pressure) dominates pathophysiology, decoupling it from CAR-associated right-heart failure markers like bilirubin. Secondary vs. Primary Valve Lesions: In functional MR/TR, CAR’s predictive strength (aHR 1.79–1.82) surpassed that in primary AR (aHR 1.98), indicating secondary lesions rely more on systemic inflammation-nutrition assessment – consistent with their frequent heart failure comorbidity. Research Implications: Rejecting a “one-size-fits-all” biomarker approach, we propose a “valve-specific biomarker matrix” (e.g., D-dimer for thrombotic risk in MS; CAR for secondary MR/TR). This precision strategy requires multicenter validation.

Perspective 3: The Inflammation-Malnutrition Axis as a Novel Therapeutic Target in VHD. Vicious Cycle Mechanism: High-CAR patients exhibit elevated CRP (median 3.32 mg/L) and hypoalbuminemia (median 39 g/L), reflecting an “inflammation → protein catabolism ↑ → malnutrition → immunosuppression → infection risk ↑ → aggravated inflammation” loop. This state prolongs post-surgical hospitalization by 28% (Citation 29). Clinical Practice Gaps: Current VHD guidelines (e.g., 2017 ESC) omit nutritional assessment, yet 7.3% of our cohort had BMI <18 kg/m^2^ with significantly higher CAR. We recommend:Routine preoperative CAR testing;For CAR >0.15^[1]^: Nutritional support (target albumin >40 g/L)^[2]^; Inflammation control (screen for infection/autoimmunity)^[3]^; Delay elective surgery until CAR improves. Economic Value: Preoperative albumin elevation by 5 g/L reduces hospitalization costs by $2100 (Citation 28), potentially offsetting testing expenses.

Perspective 4: Multimodal Risk Assessment as the Future Direction of Valvular Heart Disease Management. The integration of multimodal risk assessment represents a transformative approach to valvular heart disease (VHD) management, combining inflammatory-nutritional biomarkers (e.g., C-reactive protein-to-albumin ratio, CAR) with myocardial strain markers (e.g., NT-proBNP) to achieve earlier and more precise risk stratification. Studies demonstrate that the combined use of CAR and NT-proBNP can identify an extreme-risk subgroup with a mortality rate as high as 23.5% (eFigure 12). This “inflammation + myocardial strain” strategy outperforms traditional echocardiographic parameters like left ventricular ejection fraction (LVEF) in detecting decompensation-phase risks, as it captures both systemic metabolic derangements and hemodynamic stress before overt structural damage occurs. For instance, while LVEF reflects late-stage cardiac dysfunction, CAR and NT-proBNP synergistically reveal subclinical inflammation-driven myocardial injury (e.g., fibrosis) and volume overload, respectively. This dual-axis assessment is particularly valuable in heterogeneous VHD populations, where conditions like secondary mitral regurgitation (MR) or multivalvular disease (MVD) often involve complex interactions between systemic inflammation^[3]^, malnutrition, and ventricular remodeling. A staged implementation pathway is proposed to translate this paradigm into clinical practice:

Phase 1 (Outpatient Screening): Routine CAR and NT-proBNP testing in outpatient settings to triage high-risk patients. CAR’s cost-effectiveness (1/20th the cost of echocardiography) and NT-proBNP’s well-established prognostic value (e.g., > 1000 pg/mL predicting 1-year mortality) make them ideal for resource-limited regions.

Phase 2 (Advanced Imaging for High-Risk Patients): For those flagged by biomarker profiling, cardiac magnetic resonance imaging (CMR) can quantify myocardial fibrosis – a key determinant of irreversible ventricular dysfunction. CMR’s ability to differentiate active inflammation from scar tissue (e.g., via T1 mapping) complements biomarker data, refining surgical timing and candidacy.

Phase 3 (AI-Driven Integration): Artificial intelligence (AI) algorithms can synthesize multimodal data (clinical history, imaging, and biomarkers) to generate personalized risk scores. For example, AI-assisted analysis of echocardiographic speckle-tracking strain patterns, CMR-derived fibrosis maps, and serial NT-proBNP trends could predict left ventricular reverse remodeling potential post-valve replacement. Such integration aligns with emerging trends in VHD management, where AI-powered platforms already enhance preoperative planning (e.g., 3D valve modeling) and intraoperative guidance.

In summary, the future of VHD management lies in leveraging multimodal risk assessment to bridge the gap between pathophysiology and precision medicine. By sequentially combining accessible biomarkers, advanced imaging, and AI-driven analytics, clinicians can preemptively identify high-risk patients, optimize interventions, and ultimately improve survival and quality of life.

## Data Availability

Not applicable.
